# High expression of MRE11 correlates with poor prognosis in gastric carcinoma

**DOI:** 10.1186/s13000-019-0844-y

**Published:** 2019-06-21

**Authors:** Junqing Li, Taiqiang Su, Liang Yang, Changhua Zhang, Yulong He

**Affiliations:** 1 Digestive Disease Center,Seventh Affiliated Hospital, Sun Yat-sen University, 628 Zhenyuan Road, Shenzhen, 518000 China; 2 Department of Gastrointestinal Surgery, First Affiliated Hospital, Sun Yat-sen University, 58 Zhongshan 2nd Road, Guangzhou, 510080 China; 3 General Surgical Laboratory, First Affiliated Hospital, Sun Yat-sen University, Guangzhou, 510080 China

**Keywords:** MRE11, Gastric carcinoma, Prognosis

## Abstract

**Background:**

MRE11, a protein known to play a vital role in DNA double-strand break repair, is associated with the prognosis of a variety of tumours, but there are few studies regarding the role of MRE11 in gastric carcinoma (GC). The present study aimed to explore the clinicopathological significance and prognostic value of MRE11 expression in GC.

**Methods:**

Data from the TCGA, GEO and Oncomine databases were analysed to assess MRE11 mRNA levels in GC. The prognostic role of the level of MRE11 mRNA was examined via the Kaplan-Meier plotter. MRE11 protein expression in tumour tissues from 155 GC patients was analysed by immunohistochemistry. Relationships between MRE11 expression and clinicopathological characteristics, overall survival (OS) and recurrence-free survival (RFS) were evaluated by Cox proportional hazards regression models and Kaplan-Meier survival curves.

**Results:**

The results of bioinformatics analysis showed that MRE11 mRNA levels in GC tissues were higher than those in normal tissues (*P* < 0.01). Tissue microarray analysis showed that MRE11 protein expression was increased in GC tissues (*P* < 0.001), and MRE11 overexpression in GC tissues was significantly related to lymph node metastasis (*P* < 0.05), distant metastasis (*P* < 0.05) and tumour-node-metastasis stage (*P* < 0.05). Kaplan-Meier analyses showed that patients with GC who exhibited MRE11 overexpression had worse OS and RFS. According to Cox proportional hazards analyses, MRE11 overexpression was an independent prognostic factor for OS and RFS in these GC patients.

**Conclusions:**

MRE11 overexpression is significantly associated with poor prognosis, and MRE11 may serve as a prognostic biomarker in GC patients.

## Introduction

Gastric carcinoma (GC) is the fifth most common malignant tumour worldwide and frequently leads to death [1, 2]. Surgery is considered to be the only curative treatment, and there is no satisfactory treatment, including molecular-targeted therapy and immunotherapy, for patients with recurrent or unresectable GC [3–5]. Overall, there is a need to explore novel mechanisms of GC development to improve the prognosis of GC patients.

DNA double-strand breaks (DSBs) are a major threat to genomic integrity and cause chromosome breaks, deletions, and translocations in cancer cells [6, 7]. DSBs are repaired by the homologous recombination (HR) or non-homologous end joining (NHEJ) pathway [7, 8]. The MRE11/RAD50/NBS1 (MRN) complex plays a central role in most aspects of the cellular response to DSBs, including HR, NHEJ, telomere activity and DNA damage checkpoint activation [9–11]. As a core protein of the MRN repair complex, MRE11 may also be associated with the prognosis and development of human cancers. In addition, MRE11 protein expression was proved to be a predictive factor associated with survival following bladder cancer radiotherapy [12]. Furthermore, a randomized clinical trial showed that MRE11 deficiency is associated with improved long-term disease-free survival and overall survival (OS) in a subset of stage III colon cancer patients [13], and Yuan SS et al. showed that high MRE11 expression is associated with increased malignant behaviour in breast cancer [14]. Nonetheless, it remains unclear whether MRE11 regulates the progression and development of GC.

Therefore, in this study, we assessed MRE11 expression in GC specimens and explored its association with clinicopathologic parameters and long-term OS and recurrence-free survival (RFS) in GC patients.

## Methods

### Patients

Surgically treated GC patients (*n* = 155) with confirmed pathology at the First Affiliated Hospital of Sun Yat-sen University (FAHSYSU) between 2004 and 2007 were randomly chosen. Follow-up was terminated by December 2017. For this study, we excluded patients who received chemotherapy or other treatment before sampling or were lost during follow-up. We reviewed the patients’ clinicopathological characteristics, including gender, age, tumour size, tumour location, Bormann classification, and differentiation. We performed tumour staging for patients according to the 8th Edition of the American Joint Cancer Committee TNM classifications. Another 61 GC patients who had received surgical treatment in 2013 were randomly chosen for tissue microarray analysis.

Patient consent and ethical approval from the Institutional Review Board of FAHSYSU were obtained for this study.

### Tissue microarrays

We collected adjacent normal and GC tissues of 61 patients from the FAHSYSU Department of Pathology, and these tissues were assembled into tissue microarrays (Servicebio, Wuhan, China) for immunohistochemical (IHC) staining.

### Immunohistochemical staining

We obtained 155 paraffin-embedded GC specimens from the FAHSYSU Department of Pathology, and IHC staining of these specimens and the tissue microarrays were conducted as previously described [15] using an anti-MRE11 antibody (1:200; Sigma-Aldrich, Darmstadt, Germany).

Evaluation of the IHC results was performed by two independent investigators who were blind to the specimens, and scoring was determined using a semi-quantitative method [16]. Samples in which more than 10% of the tumour cells were stained were considered positive. The staining intensity was defined as follows: 0 (negative), 1 (weak), 2 (moderate), and 3 (strong). Negative and weak staining was considered to indicate low MRE11 expression, and moderate and strong staining was considered to indicate high MRE11 expression.

### Bioinformatics analysis

We downloaded RNA-Seq data for GC from The Cancer Genome Atlas (TCGA), GEO (GSE139911) and Oncomine databases. We analysed the prognostic role of MRE11 mRNA levels using the Kaplan-Meier plotter.

### Statistical analysis

Statistical analyses were performed using SPSS 17.0 (IBM, NY, USA). The chi-square test was employed for numerical data. Survival curves were generated using the Kaplan-Meier estimator. Hazard ratios (HRs) and 95% confidence intervals (CIs) were computed from univariate and multivariate Cox proportional hazards regression models to examine associations between prognosis and clinicopathological characteristics. *P* values less than 0.05 were considered statistically significant.

## Results

### Overexpression of MRE11 mRNA in GC tissues

**Fig. 1:**
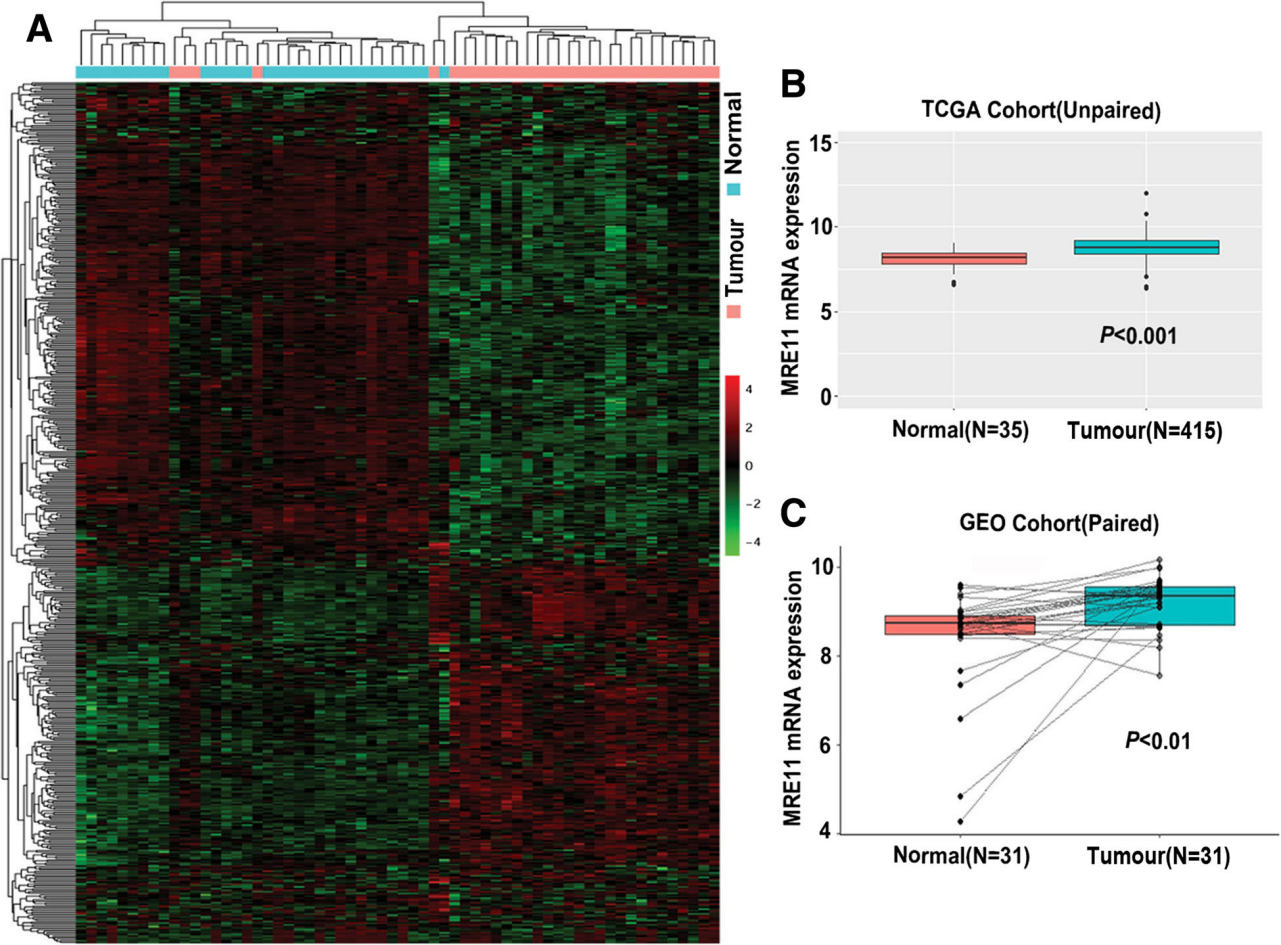
High MRE11 expression in GC. **a** Analysis of TCGA data in a heat map. **b** MRE11 mRNA expression in unpaired GC and normal tissues from the TCGA database. **c** MRE11 mRNA expression in paired GC and normal tissues from the GEO database

Data from TCGA showed many genes to be up- or downregulated in GC samples relative to their expression in normal samples, with the MRE11 gene being significantly overexpressed in the former (Fig. 1a). Further analysis of unpaired GC and normal tissues from TCGA also indicated markedly upregulated expression of MRE11 mRNA in GC tissues (*P* < 0.001; Fig. 1b). The same results were found for paired GC and normal tissues from the GEO cohort (*P* < 0.01; Fig. 1c), and Oncomine cohort data were consistent (Table 1). Taken together, these findings indicate that MRE11 mRNA expression was upregulated in GC tissues.

**Table 1:** Oncomine analysis of MRE11 expression in GC (total of 5 GC cohorts)

Cohort	Sample (n)	t Test	Fold change	*P*
D’Errico et al. [17]	Gastric Mixed Adenocarcinoma (4) vs Normal (31)	6.160	2.953	3.34E-6
Cho et al. [18]	Diffuse Gastric Adenocarcinoma (31) vs Normal (19)	4.419	1.308	2.84E-5
	Gastric Mixed Adenocarcinoma (10) vs Normal (19)	3.969	1.383	7.56E-4
	Gastric Adenocarcinoma (4) vs Normal (19)	2.401	1.386	0.042
	Gastric Intestinal-Type Adenocarcinoma (20) vs Normal (19)	2.340	1.200	0.013
Wang et al. [19]	Gastric Cancer (3) vs Normal (12)	2.958	1.668	0.003
Cui et al. [20]	Gastric Cancer (80) vs Normal (80)	1.882	1.283	0.031
Deng et al. [21]	Gastric Intestinal Type Adenocarcinoma (44) vs Normal (17)	2.575	1.022	0.007
	Diffuse Gastric Adenocarcinoma (13) vs Normal (17)	2.163	1.011	0.018

### Associations of MRE11 expression with clinical parameters in GC

**Fig. 2:**
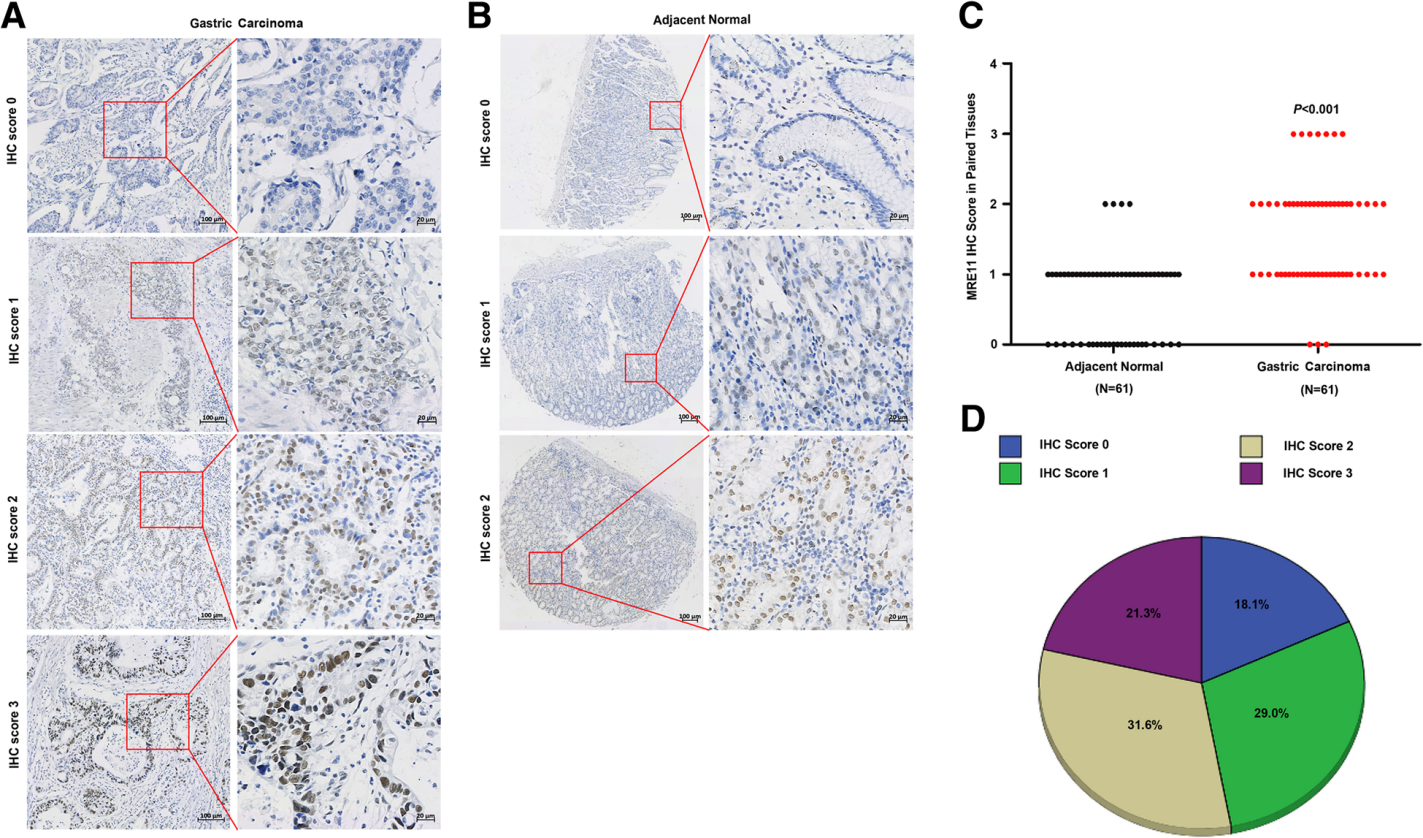
MRE11 protein expression in GC tissues and adjacent normal tissues. **a** IHC staining of the MRE11 protein in GC tissues. **b** IHC staining of the MRE11 protein in adjacent normal tissues. **c** MRE11 protein expression was higher in GC specimens than in adjacent normal tissues, as indicated by IHC staining. **d** Percentage of patients with GC according to MRE11 protein expression as indicated by IHC scoring

To evaluate the relationship between MRE11 expression and clinicopathological characteristics in GC, we performed IHC staining to detect MRE11 expression in 155 paraffin-embedded GC specimens. As shown in Fig. 2, the MRE11 protein was mainly distributed in the nucleus. MRE11 expression was further measured using tissue microarrays containing adjacent normal and GC tissues from 61 patients, and MRE11 protein expression was found to be significantly higher in GC tissues than in adjacent normal tissues (Fig. 2c). As shown in Fig. 2d, there were 28 cases (18.1%) with an IHC score of 0, 45 (29.0%) with an IHC score of 1, 49 (31.6%) with an IHC score of 2, and 33 (21.3%) with an IHC score of 3. IHC scores of 0 and 1 were defined as low MRE11 expression (47.1%, 73/155); IHC scores of 2 and 3 were defined as high MRE11 expression (52.9%, 82/155).

The relationships between MRE11 expression and clinicopathological parameters are summarized in Table 2. MRE11 overexpression in GC tissues was significantly related to lymph node metastasis (*P* < 0.05), distant metastasis (*P* < 0.05) and tumour-node-metastasis (TNM) stage (*P* < 0.05).

**Table 2:** Associations of MRE11 expression with clinical parameters in GC

Characteristic	No.	MRE11 Expression		χ^2^	P
		Low(*N* = 73)	High(*N* = 82)	Value	Value
Age		59.84 ± 10.87	56.92 ± 13.24		
<60y	78	35	43	0.312	0.576
≥60y	77	38	39		
Gender					
Man	99	48	51	0.212	0.645
Female	56	25	31		
Tumour location					
Proximal	33	14	19	2.575	0.462
Middle	29	17	12		
Distal	58	28	30		
More than 2	35	14	21		
Tumour size					
< 5 cm	68	34	34	0.410	0.522
≥5 cm	87	39	48		
Histologic type					
Adenocarcinoma (NOS)	132	63	69	1.868	0.600
Signet ring	9	4	5		
Mucinous	12	6	6		
Undifferentiated	2	0	2		
Bornmann classification					
I	8	2	6	2.107	0.550
II	34	18	16		
III	97	45	52		
IV	16	8	8		
Differentiation					
Well	3	2	1	1.517	0.468
Moderate	35	19	16		
Poor	117	52	65		
Depth of invasion					
T1	15	10	5	4.146	0.246
T2	13	8	5		
T3	84	36	48		
T4	43	19	24		
Lymph node metastasis					
N0	45	26	19	8.269	0.041
N1	54	25	29		
N2	23	5	18		
N3	33	17	16		
Distant metastasis					
M0	126	65	61	5.450	0.020
M1	29	8	21		
Tumour-Node-Metastasis stage					
I + II	49	30	19	5.740	0.017
III + IV	106	43	63		
CEA level (μg/L)					
< 5	135	64	71	0.041	0.840
≥5	20	9	11		

### High MRE11 expression predicted worse survival in GC

**Fig. 3:**
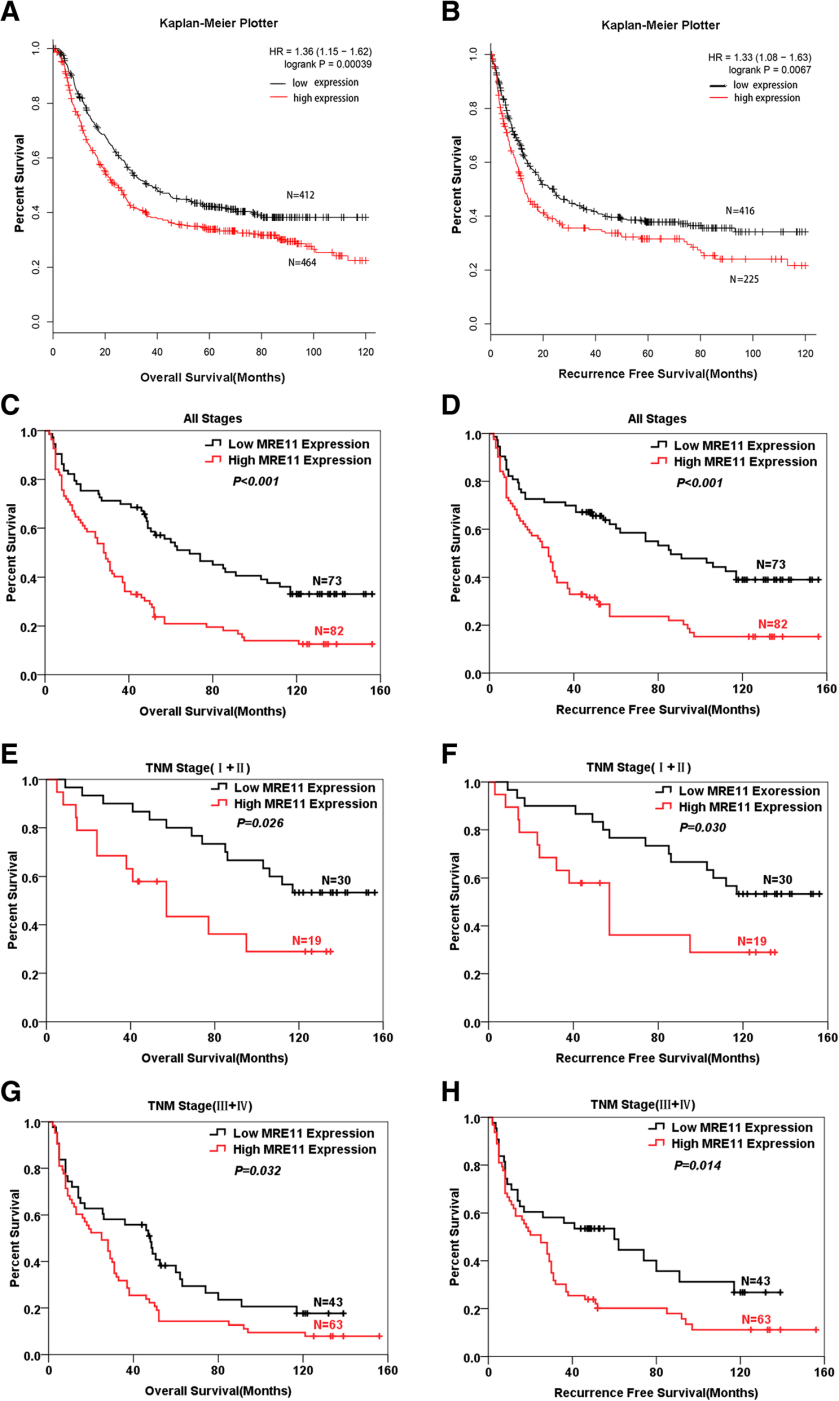
Patients with high MRE11 expression had a poor prognosis. **a** and **b** Kaplan-Meier Plotter OS and RFS curves for GC patient groups with low and high MRE11 mRNA levels. **c** and **d** OS and RFS of patients with high MRE11 overexpression were worse than those of patients with low MRE11 expression. **e** and **f** Patients with high MRE11 expression had worse OS and RFS than did those with low MRE11 expression in early GC (TNM stages I and II). **g** and **h** High MRE11 expression in GC tissues predicted worse OS and RFS in patients with advanced GC (TNM stages III and IV)

To define the prognostic role of MRE11 expression in GC, we first analysed data using the Kaplan-Meier plotter. Patients with high levels of MRE11 mRNA had worse OS (Fig. 3a) and RFS (Fig. 3b) than those with low MRE11 levels. Next, the prognostic value of MRE11 protein expression in our cohort was assessed by Kaplan-Meier analysis. The follow-up period of the 155 GC patients ranged from 2 to 156 months, with a mean survival time of 62.56 ± 4.64 months. The mean survival times of patients with low and high MRE11 expression were 81.83 ± 7.06 and 45.37 ± 5.47 months, respectively. The combined 5-year OS rate was 36.6%; the 5-year OS rate was 54.1% in the low MRE11 expression group and 20.9% in the high MRE11 expression group. Our data indicated that high MRE11 expression was associated with worse OS (*P* < 0.001, Fig. 3c) and RFS (*P* < 0.001, Fig. 3d). Furthermore, we defined the prognostic value of MRE11 expression in early (TNM stages I and II) and advanced (TNM stages III and IV) GC, and the results showed that high MRE11 expression was associated with worse OS (*P* < 0.05, Fig. 3e and g) and RFS (*P* < 0.05, Fig. 3f and h) in both early and advanced disease.

We also performed univariate and multivariate analyses to assess the ability of MRE11 expression to predict the prognosis of patients with GC. As shown in Table 3, univariate analysis revealed that certain clinical variables were significantly associated with OS, with multivariate analysis demonstrating that MRE11 expression (*P* < 0.01) was an independent predictor of OS in GC patients. In addition, MRE11 expression was an independent predictor of RFS (*P* < 0.01) in GC patients (Table 4). Taken together, our results indicate that MRE11 expression was an independent prognostic factor for the GC patients investigated and that MRE11 might serve as a molecular marker for GC prognosis.

**Table 3:** Cox proportional-hazard regression analysis for Overall Survival

Characteristic	Univariate analysis				Multivariate analysis			
	*P*-Value	HR	95.0% CI for Exp(B)		P-Value	HR	95.0% CI for Exp(B)	
			Lower	Upper			Lower	Upper
Gender	0.016	1.582	1.089	2.300	0.029	1.546	1.045	2.286
Age	0.325	0.833	0.580	1.198				
Tumour location	0.497	1.064	0.889	1.274				
Tumour size	0.001	1.898	1.303	2.763				
Histologic type	0.335	1.174	0.847	1.628				
Bornmann classification	0.008	1.460	1.103	1.932				
Differentiation	0.034	1.550	1.033	2.326				
Depth of invasion	0.000	1.757	1.382	2.235				
Lymph node metastasis	0.000	1.521	1.289	1.794				
Distant metastasis	0.000	3.509	2.267	5.429				
Tumour-Node-Metastasis stage	0.000	2.134	1.698	2.681	0.016	1.735	1.107	2.719
CEA	0.019	1.832	1.104	3.040				
MRE11 expression	0.000	2.069	1.422	3.012	0.001	1.900	1.297	2.782

**Table 4:** Cox proportional-hazard regression analysis for Recurrence Free Survival

Characteristic	Univariate analysis			Multivariate analysis				
	P-Value	HR	95.0% CI for Exp(B)		P-Value	HR	95.0% CI for Exp(B)	
			Lower	Upper			Lower	Upper
Gender	0.045	1.497	1.009	2.221				
Age	0.404	0.850	0.581	1.245				
Tumour location	0.901	0.988	0.820	1.191				
Tumour size	0.006	1.735	1.173	2.565				
Histologic type	0.464	1.144	0.798	1.641				
Bornmann classification	0.025	1.402	1.043	1.884				
Differentiation	0.036	1.589	1.030	2.452				
Depth of invasion	0.001	1.526	1.199	1.940				
Lymph node metastasis	0.001	1.331	1.116	1.586				
Distant metastasis	0.000	3.108	1.965	4.915				
Tumour-Node-Metastasis stage	0.000	1.928	1.527	2.433	0.005	1.969	1.232	3.147
CEA	0.042	1.744	1.021	2.979				
MRE11 expression	0.000	2.220	1.489	3.309	0.001	2.041	1.358	3.068

## Discussion

The results of this study show that MRE11 overexpression is significantly associated with poor prognosis and that MRE11 is a potential prognostic biomarker in patients with GC. First, by analysing TCGA, GEO and Oncomine data, we demonstrated that the MRE11 mRNA levels were significantly higher in GC tissues than in normal tissues. Second, using the Kaplan-Meier plotter, we found that patients with high MRE11 expression had poor OS and RFS. Third, we proved that MRE11 protein expression was significantly higher in GC tissues than in adjacent normal tissues, as based on tissue microarrays derived from 61 patients. Furthermore, IHC staining of tissues from 155 GC patients verified that MRE11 overexpression was associated with poor OS and RFS, a finding that is consistent with that of a previous study [22]. Finally, multivariate Cox regression analysis demonstrated MRE11 expression to be an independent prognostic factor for OS and RFS in GC patients.

The MRE11 gene encodes a nuclear protein involved in HR, telomere length maintenance, and DSB repair, and the MRN complex is required for NHEJ [6–8, 11]. Previous studies have reported that deficiency of the MRN complex may sensitize cancer cells to treatment with PARP inhibitors and might serve as a predictive biomarker for the efficacy of PARP inhibitor therapy [23–25]. MRE11 protein expression is also associated with the prognosis and development of human cancers, such as bladder cancer, colon cancer, and breast cancer [12–14]. Altan B et al. [22] found that high expression of MRE11 is associated with poor OS in GC, and we further proved that MRE11 overexpression is associated with poor RFS and is an independent prognostic factor for OS and RFS in GC. Some studies have suggested that high expression of MRE11 in tumour cells enhances DSB repair, leading to increased local recurrence and reduced survival rates [14, 26].

The findings of this study also demonstrate that MRE11 overexpression is significantly related to lymph node metastasis, distant metastasis and TNM stage. However, we did not explore the molecular mechanism of MRE11 in GC, which is a limitation of this study. Previous studies have shown that MRE11 overexpression in breast cancer cells leads to cell proliferation by stimulating STAT3 signalling and enhances migration and invasion capabilities through activation of MMP-2 and MMP-9 [14]. In addition, activation of the STAT3-MMP axis has been reported to promote cancer cell invasiveness and metastasis [27–29]. Based on these results, we can conclude that the STAT3-MMP axis may be involved in the molecular mechanism of MRE11 in GC. In future work, we will verify the role of this molecular pathway in GC. As tumour cells overexpressing MRE11 exhibit a poor response to radiotherapy and chemotherapy and MRE11 deficiency leads to inhibition of DSB repair and enhances radio- and chemosensitivity in tumour cells [12, 14, 30–32], MRE11 is a potential target for increasing radio- and chemosensitivity.

In conclusion, MRE11 overexpression is significantly associated with poor prognosis, and MRE11 may act as a prognostic biomarker in patients with GC. MRE11 may also serve as a target molecule for chemoradiotherapy.

## Data Availability

The data from our study are available from the corresponding authors upon reasonable request.

## Abbreviations

CIs: Confidence intervals
DSBs: DNA double-strand breaks
HR: Homologous recombination
HRs: Hazard ratios
IHC: immunohistochemical
MRN: MRE11/RAD50/NBS1
NHEJ: Non-homologous end joining
OS: Overall survival
RFS: Recurrence-free survival

## References

[1] Torre LA, Bray F, Siegel RL, Ferlay J, Lortet-Tieulent J, Jemal A (2015). Global cancer statistics, 2012. CA Cancer J Clin.

[2] Ferlay J, Soerjomataram I, Dikshit R, Eser S, Mathers C, Rebelo M (2015). Cancer incidence and mortality worldwide: sources, methods and major patterns in GLOBOCAN 2012. Int J Cancer.

[3] Cidon EU, Ellis SG, Inam Y, Adeleke S, Zarif S, Geldart T (2013). Molecular targeted agents for gastric cancer: a step forward towards personalized therapy. Cancers (Basel).

[4] Song Z, Wu Y, Yang J, Yang D, Fang X (2017). Progress in the treatment of advanced gastric cancer. Tumour Biol.

[5] Vrána D, Matzenauer M, Neoral Č, Aujeský R, Vrba R, Melichar B (2018). From tumor immunology to immunotherapy in gastric and esophageal Cancer. Int J Mol Sci.

[6] Powell SN, Bindra RS (2009). Targeting the DNA damage response for cancer therapy. DNA Repair (Amst).

[7] Rodgers K, McVey M (2016). Error-prone repair of DNA double-Strand breaks. J Cell Physiol.

[8] Helleday T, Lo J, van Gent DC, Engelward BP (2007). DNA double-strand break repair: from mechanistic understanding to cancer treatment. DNA Repair (Amst).

[9] Assenmacher N, Hopfner KP (2004). MRE11/RAD50/NBS1: complex activities. Chromosoma.

[10] Williams RS, Williams JS, Tainer JA (2007). Mre11-Rad50-Nbs1 is a keystone complex connecting DNA repair machinery, double-strand break signaling, and the chromatin template. Biochem Cell Biol.

[11] Kavitha CV, Choudhary B, Raghavan SC, Muniyappa K (2010). Differential regulation of MRN (Mre11-Rad50-Nbs1) complex subunits and telomerase activity in cancer cells. Biochem Biophys Res Commun.

[12] Choudhury A, Nelson LD, Teo MT, Chilka S, Bhattarai S, Johnston CF (2010). MRE11 expression is predictive of cause-specific survival following radical radiotherapy for muscle-invasive bladder cancer. Cancer Res.

[13] Pavelitz T, Renfro L, Foster NR, Caracol A, Welsch P, Lao VV (2014). MRE11-deficiency associated with improved long-term disease free survival and overall survival in a subset of stage III colon cancer patients in randomized CALGB 89803 trial. PLoS One.

[14] Yuan SS, Hou MF, Hsieh YC, Huang CY, Lee YC, Chen YJ (2012). Role of MRE11 in cell proliferation, tumor invasion, and DNA repair in breast cancer. J Natl Cancer Inst.

[15] Yang L, Chen Z, Xiong W, Ren H, Zhai E, Xu K (2018). High expression of SLC17A9 correlates with poor prognosis in colorectal cancer. Hum Pathol.

[16] Zhai E, Liang W, Lin Y, Huang L, He X, Cai S (2018). HSP70/HSP90-organizing protein contributes to gastric Cancer progression in an autocrine fashion and predicts poor survival in gastric Cancer. Cell Physiol Biochem.

[17] D'Errico M, de Rinaldis E, Blasi MF, Viti V, Falchetti M, Calcagnile A (2009). Genome-wide expression profile of sporadic gastric cancers with microsatellite instability. Eur J Cancer.

[18] Cho JY, Lim JY, Cheong JH, Park YY, Yoon SL, Kim SM (2011). Gene expression signature-based prognostic risk score in gastric cancer. Clin Cancer Res.

[19] Wang Q, Wen YG, Li DP, Xia J, Zhou CZ, Yan DW (2012). Upregulated INHBA expression is associated with poor survival in gastric cancer. Med Oncol.

[20] Cui J, Chen Y, Chou WC, Sun L, Chen L, Suo J (2011). An integrated transcriptomic and computational analysis for biomarker identification in gastric cancer. Nucleic Acids Res.

[21] Deng N, Goh LK, Wang H, Das K, Tao J, Tan IB (2012). A comprehensive survey of genomic alterations in gastric cancer reveals systematic patterns of molecular exclusivity and co-occurrence among distinct therapeutic targets. Gut.

[22] Altan B, Yokobori T, Ide M, Bai T, Yanoma T, Kimura A (2016). High expression of MRE11-RAD50-NBS1 is associated with poor prognosis and Chemoresistance in gastric Cancer. Anticancer Res.

[23] Oplustilova L, Wolanin K, Mistrik M, Korinkova G, Simkova D, Bouchal J (2012). Evaluation of candidate biomarkers to predict cancer cell sensitivity or resistance to PARP-1 inhibitor treatment. Cell Cycle.

[24] Koppensteiner R, Samartzis EP, Noske A, von Teichman A, Dedes I, Gwerder M (2014). Effect of MRE11 loss on PARP-inhibitor sensitivity in endometrial cancer in vitro. PLoS One.

[25] Brandt S, Samartzis EP, Zimmermann AK, Fink D, Moch H, Noske A (2017). Lack of MRE11-RAD50-NBS1 (MRN) complex detection occurs frequently in low-grade epithelial ovarian cancer. BMC Cancer.

[26] LeScodan R, Cizeron-Clairac G, Fourme E, Meseure D, Vacher S, Spyratos F (2010). DNA repair gene expression and risk of locoregional relapse in breast cancer patients. Int J Radiat Oncol Biol Phys.

[27] Huang C, Cao J, Huang KJ, Zhang F, Jiang T, Zhu L (2006). Inhibition of STAT3 activity with AG490 decreases the invasion of human pancreatic cancer cells in vitro. Cancer Sci.

[28] Xie TX, Wei D, Liu M, Gao AC, Ali-Osman F, Sawaya R (2004). Stat3 activation regulates the expression of matrix metalloproteinase-2 and tumor invasion and metastasis. Oncogene.

[29] Wu X, Yan Q, Zhang Z, Du G, Wan X (2012). Acrp30 inhibits leptin-induced metastasis by downregulating the JAK/STAT3 pathway via AMPK activation in aggressive SPEC-2 endometrial cancer cells. Oncol Rep.

[30] Kuroda S, Fujiwara T, Shirakawa Y, Yamasaki Y, Yano S, Uno F (2010). Telomerase-dependent oncolytic adenovirus sensitizes human cancer cells to ionizing radiation via inhibition of DNA repair machinery. Cancer Res.

[31] Deng R, Tang J, Ma JG, Chen SP, Xia LP, Zhou WJ (2011). PKB/Akt promotes DSB repair in cancer cells through upregulating Mre11 expression following ionizing radiation. Oncogene.

[32] Zhang J, Xin X, Chen Q, Xie Z, Gui M, Chen Y (2012). Oligomannurarate sulfate sensitizes cancer cells to doxorubicin by inhibiting atypical activation of NF-kappaB via targeting of Mre11. Int J Cancer.

